# Supplementary material for: A Permian fish reveals widespread distribution of neopterygian-like jaw suspension
Source: eLife. 2022 May 17;11:e58433. doi: 10.7554/eLife.58433 (PMC9345605; doi:10.7554/eLife.58433)
Supplement: Transparent reporting form [file elife-58433-transrepform1.docx]

***eLife’s* transparent reporting form**

We encourage authors to provide detailed information *within their submission* to facilitate the interpretation and replication of experiments. Authors can upload supporting documentation to indicate the use of appropriate reporting guidelines for health-related research (see [EQUATOR Network](http://www.equator-network.org/%20)), life science research (see the [BioSharing Information Resource](https://biosharing.org/)), or the [ARRIVE guidelines](http://www.plosbiology.org/article/info:doi/10.1371/journal.pbio.1000412) for reporting work involving animal research. Where applicable, authors should refer to any relevant reporting standards documents in this form.

If you have any questions, please consult our Journal Policies and/or contact us: [editorial@elifesciences.org](mailto:editorial@elifesciences.org).

**Sample-size estimation**

- You should state whether an appropriate sample size was computed when the study was being designed
- You should state the statistical method of sample size computation and any required assumptions
- If no explicit power analysis was used, you should describe how you decided what sample (replicate) size (number) to use

Please outline where this information can be found within the submission (e.g., sections or figure legends), or explain why this information doesn’t apply to your submission:

This study deals with novel anatomical data deriving from unique paleontological specimens. Both specimens attributed to †*Brachydegma caelatum* were included in this study. Additional specimens of vertebrate fossil and extant taxa (sample sizes equal to 1–2) were taken into account for comparison. Detailed information on study material can be found in the ‘Materials and methods’ section of our manuscript.

**Replicates**

- You should report how often each experiment was performed
- You should include a definition of biological versus technical replication
- The data obtained should be provided and sufficient information should be provided to indicate the number of independent biological and/or technical replicates
- If you encountered any outliers, you should describe how these were handled
- Criteria for exclusion/inclusion of data should be clearly stated
- High-throughput sequence data should be uploaded before submission, with a private link for reviewers provided (these are available from both GEO and ArrayExpress)

Please outline where this information can be found within the submission (e.g., sections or figure legends), or explain why this information doesn’t apply to your submission:

μCT imaging of fossil and extant taxa was performed once for each specimen. Relevant information on the parameters used for μCT imaging is given in the ‘materials and methods’ section. We also provide raw tomographic volumes and detailed μCT parameter logs for both†*Brachydegma* specimens, to ensure the reproducibility of our results and further examination of our anatomical interpretations.

The final phylogenetic trees presented in this work resulted from numerous iterations of various algorithms. The parameters for both parsimony and Bayesian analyses are outlined in detail in the ‘materials and methods section’. We provide all relevant data (phylogenetic matrix, trees, new character descriptions and matrix alterations) that would allow for the reproducibility of our results in the supplement.

**Statistical reporting**

- Statistical analysis methods should be described and justified
- Raw data should be presented in figures whenever informative to do so (typically when N per group is less than 10)
- For each experiment, you should identify the statistical tests used, exact values of N, definitions of center, methods of multiple test correction, and dispersion and precision measures (e.g., mean, median, SD, SEM, confidence intervals; and, for the major substantive results, a measure of effect size (e.g., Pearson's r, Cohen's d)
- Report exact p-values wherever possible alongside the summary statistics and 95% confidence intervals. These should be reported for all key questions and not only when the p-value is less than 0.05.

Please outline where this information can be found within the submission (e.g., sections or figure legends), or explain why this information doesn’t apply to your submission:

μCT imaging of fossil and extant taxa was performed once for each specimen. Relevant information on the parameters used for μCT imaging is given in the ‘materials and methods’ section. We also provide raw tomographic volumes and detailed μCT parameter logs in the supplement for both †*Brachydegma* specimens, to ensure the reproducibility of our results and further examination of our anatomical interpretations.

(For large datasets, or papers with a very large number of statistical tests, you may upload a single table file with tests, Ns, etc., with reference to sections in the manuscript.)

**Group allocation**

- Indicate how samples were allocated into experimental groups (in the case of clinical studies, please specify allocation to treatment method); if randomization was used, please also state if restricted randomization was applied
- Indicate if masking was used during group allocation, data collection and/or data analysis

Please outline where this information can be found within the submission (e.g., sections or figure legends), or explain why this information doesn’t apply to your submission:

This does not apply to our submission, as it does not include relevant statistical or clinical tests.

**Additional data files (“source data”)**

- We encourage you to upload relevant additional data files, such as numerical data that are represented as a graph in a figure, or as a summary table
- Where provided, these should be in the most useful format, and they can be uploaded as “Source data” files linked to a main figure or table
- Include model definition files including the full list of parameters used
- Include code used for data analysis (e.g., R, MatLab)
- Avoid stating that data files are “available upon request”

Please indicate the figures or tables for which source data files have been provided:

We add all files employed in our phylogenetic analyses (matrix, and resulting trees) as supplement to our manuscript.

Additional data will be deposited in dryad. These include: 1) raw tomographic volume files and scanning parameter logs for both *Brachydegma* specimens; 2) digital anatomical surface models in .ply for *Brachydegma*, Parasemionotid, *Acipenser*, *Pteronisculus*.
